# N-acetyl ornithine deacetylase is a moonlighting protein and is involved in the adaptation of *Entamoeba histolytica* to nitrosative stress

**DOI:** 10.1038/srep36323

**Published:** 2016-11-03

**Authors:** Preeti Shahi, Meirav Trebicz-Geffen, Shruti Nagaraja, Rivka Hertz, Sharon Alterzon-Baumel, Karen Methling, Michael Lalk, Mohit Mazumder, Gourinath Samudrala, Serge Ankri

**Affiliations:** 1 Department of Molecular Microbiology, Ruth and Bruce Rappaport Faculty of Medicine, Technion, P.O.B. 9649, 31096 Haifa Israel; 2 University of Greifswald, Institute of Biochemistry, Greifswald, Germany; 3 Jawaharlal Nehru University School of Life Sciences, New Delhi, India

## Abstract

Adaptation of the *Entamoeba histolytica* parasite to toxic levels of nitric oxide (NO) that are produced by phagocytes may be essential for the establishment of chronic amebiasis and the parasite’s survival in its host. In order to obtain insight into the mechanism of *E. histolytica*’s adaptation to NO, *E. histolytica* trophozoites were progressively adapted to increasing concentrations of the NO donor drug, S-nitrosoglutathione (GSNO) up to a concentration of 110 μM. The transcriptome of NO adapted trophozoites (NAT) was investigated by RNA sequencing (RNA-seq). N-acetyl ornithine deacetylase (NAOD) was among the 208 genes that were upregulated in NAT. NAOD catalyzes the deacetylation of N-acetyl-L-ornithine to yield ornithine and acetate. Here, we report that NAOD contributes to the better adaptation of the parasite to nitrosative stress (NS) and that this function does not depend on NAOD catalytic activity. We also demonstrated that glyceraldehyde 3-phosphate dehydrogenase (GAPDH) is detrimental to *E. histolytica* exposed to NS and that this detrimental effect is neutralized by NAOD or by a catalytically inactive NAOD (mNAOD). These results establish NAOD as a moonlighting protein, and highlight the unexpected role of this metabolic enzyme in the adaptation of the parasite to NS.

Intestinal infections are a global medical problem and diarrheal disease is one of the main causes of childhood morbidity and mortality. *Entamoeba histolytica* is a protozoan parasite and the causal agent of amebiasis, the second most common cause of death from parasitic disease worldwide after malaria (at least 100,000 deaths each year). According to the World Health Organization, amebic dysentery affects 50 million people in India, Southeast Asia, Africa, and Latin America. Since poor sanitary conditions and unsafe hygiene practices exist in many parts of the world, the main mode of transmission of amebiasis is the ingestion of food and/or water that is contaminated with feces and *E. histolytica* cysts. *E. histolytica* trophozoites are non-pathogenic commensals in 90% of infected individuals (asymptomatic amebiasis). For unknown reasons, some of these trophozoites can invade the intestinal mucosa, cause dysentery, and migrate to the liver where they produce abscesses (extraintestinal amebiasis). In the large intestine*, E. histolytica* is exposed to nanomolar concentrations of nitric oxide (NO) that is produced in intestinal epithelial cells by constitutive NO synthase (NOS)^1^ and as an intermediate in denitrification by the intestinal microbiota^2^. Although exposure to low NO concentrations is insufficient to kill the parasite^3^, these low concentrations may strengthen its resistance to high NO concentrations. Amebiasis is characterized by acute inflammation of the intestine with the release of cytokines, such as tumor necrosis factor α (TNFα), interleukin 8 (IL-8), interferon gamma (IFN-γ), and interleukin β (IL-1β), and the generation of micromolar concentrations of reactive oxygen species (ROS) and reactive nitrogen species (RNS) from activated cells of the host’s immune system (for a recent review see ref. 4). NO in micromolar concentrations is cytotoxic for *E. histolytica*, and this cytotoxicity is implemented by S-nitrosylation of key metabolic enzymes and by fragmenting the endoplasmic reticulum (ER)^5^. NO also inhibits cysteine proteases (CPs)^6^, which are involved in differentiation, amino acid anabolism, inactivation of the host inflammatory response, lysosomal transport, and invasion of the host’s tissues (for a recent review see ref. 7). NO can also regulate the activity and function of proteins by S-nitrosylation of their cysteine residues^8^. Recently, we completed a high-throughput proteomic analysis of S-nitrosylated (SNO) proteins in NO-exposed *E. histolytica* using resin-assisted capture of SNO proteins (SNO-RAC)^3^, and found that SNO proteins are involved in glycolysis, translation, protein transport, and virulence. It has been reported that *E. histolytica* can adapt to various stresses^9^,^10^,^11^,^12^,^13^. Nevertheless, information on the ability of the parasite to adapt to progressive increases in the intestinal NO concentration, which may occur in patients with inflammation of the large intestine^1^ or during the establishment of amebiasis^14^, is lacking. In order to obtain insight into the mechanism of NO resistance in *E. histolytica*, NO adapted trophozoites (NAT) were produced *in vitro* by stepwise exposure to increasing amounts of S-nitrosoglutathione (GSNO), an NO donor, which is the S-nitrosated derivative of glutathione. Phenotypic analysis of NAT show that they are more resistant to activated macrophages and their binding to target cells is substantially stronger that of trophozoites which were exposed to an acute nitrosative stress (TEANS)^15^. However the growth rate and sensitivity of NAT to oxidative stress (OS) is less than those of control trophozoites. In this report, we present the results of a comparative transcriptome analysis of control untreated trophozoites and NAT. We also revealed that N-acetyl-L-ornithine deacetylase (NAOD) is a moonlighting protein which has been recruited by *E. histolytica* for its adaptation to NO.

## Results

### *E. histolytica* exhibits an adaptive response to GSNO

**Figure 1:**
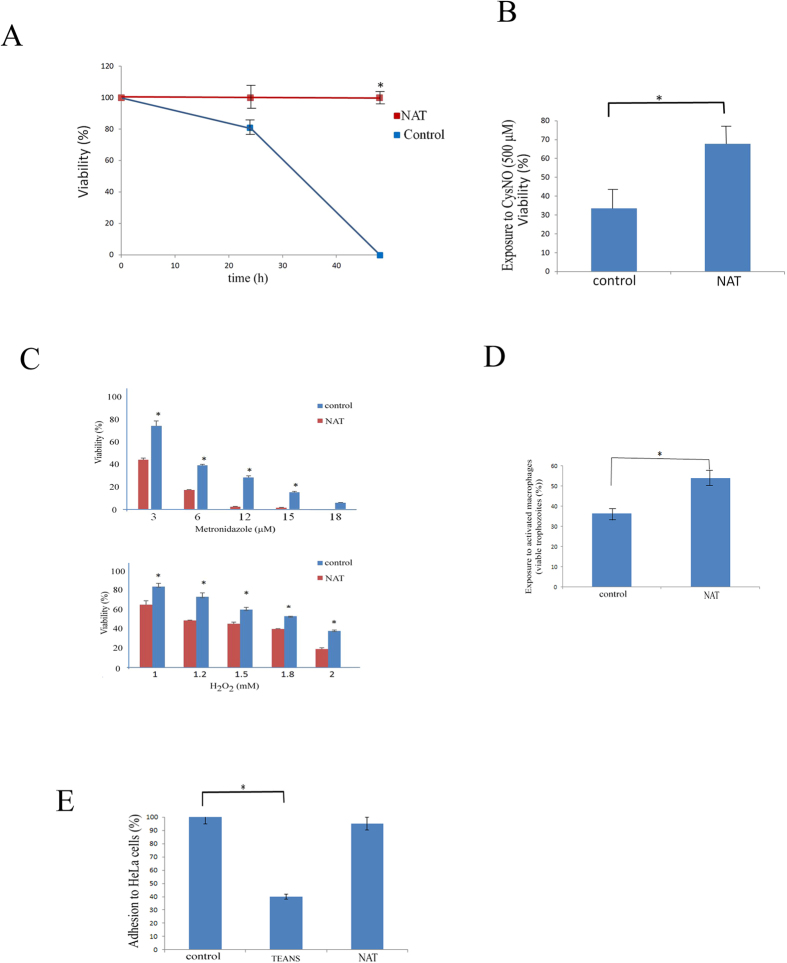
Characterization of NO-adapted trophozoites. (**A**) Viability of control trophozoites and NAT cultivated in presence of GSNO (110 μM). Control trophozoites and NAT. The number of living trophozoites was determined after 24 h and 48 h of culture. Data are expressed as the mean ± standard deviation of three independent experiments that were repeated twice. *p ≤ 0.05 and is the significance of the difference between the viability of the control trophozoites and NAT after 48 h of culture according to the results of an unpaired Student’s t-test. (**B**) Viability of control and GSNO trophozoites exposed to CysNO (500 μM). Data are expressed as the mean ± SD of three independent experiments that were repeated twice. *p ≤ 0.05 and is the significance of the difference between the viability of the control trophozoites and NAT according to the results of an unpaired Student’s t-test. (**C**) Viability of NAT exposed to metronidazole or to H_2_O_2_. Data are expressed as the mean ± SD of three independent experiments that were repeated twice. *p ≤ 0.05 and is the significance of the difference between the viability of the control trophozoites and NAT according to the results of an unpaired Student’s t-test. (**D**) Viability of control *E. histolytica* trophozoites and NO-adapted trophozoites (NAT) after exposure to activated macrophages. Data are expressed as the mean ± SD of three independent experiments that were repeated twice. *p ≤ 0.05 and is the significance of the difference between the viability of the control trophozoites and NAT according to the results of an unpaired Student’s t-test. (**E**) Adhesion of *E. histolytica* control trophozoites and NO-adapted trophozoites (NAT) to HeLa cells. The amount of adhesion of the control trophozoites was set at 100%. Data are expressed as the mean ± SD of three independent experiments that were performed in triplicate. The adhesion of the control trophozoites was significantly different (p ≤ 0.05) to the adhesion of trophozoites that were exposed to GSNO according to the results of an unpaired Student’s t-test. No significant difference between the adhesion of control trophozoites and the NAT was detected.

In order to determine whether *E. histolytica* can be adapted to survive in micromolar concentrations of NO, *E. histolytica* trophozoites were exposed to increasing GSNO concentrations by stepwise additions of GSNO to the culture medium to a final concentration of 110 μM over one month. Attempts to adapt the parasite to higher GSNO concentrations were unsuccessful. Trophozoites that have never been exposed to GSNO (control trophozoites) died within 48 hours when exposed to 110 μM GSNO (Fig. 1A). In order to determine whether adaptation of *E. histolytica* to NO has an effect on its growth rate, we measured the generation time of NAT. The generation time of control trophozoites was 50% of that of NAT (10.2 ± 0.5 hours versus 20 ± 1 hours; p < 0.005). NAT were also resistant to 500 μM S-nitroso-L-cysteine (CysNO), another NO donor drug (Fig. 1B). NAT were also investigated for their resistance to H_2_O_2_ and metronidazole: NAT were significantly more sensitive to H_2_O_2_ and metronidazole than control trophozoites (Fig. 1C).

IFN_γ_ and LPS-activated mouse macrophages use RNS to kill *E. histolytica* trophozoites^16^. It has been recently determined that activated mouse macrophages produced significant amount of NO (96 amol/(min. cell))^17^. When we compared the cytotoxic activity of activated mouse macrophages on control trophozoites and NAT, we found that NAT are more resistant to activated macrophages than the control trophozoites (Fig. 1D).

The ability of TEANS to adhere to mammalian cells is impaired^15^ (Fig. 1E). We determined the ability of NAT to adhere to HeLa cells. Surprisingly, we found that the ability of NAT and control trophozoites to adhere to mammalian cells was similar (Fig. 1E).

### Transcriptome analysis of NAT

**Figure 2:**
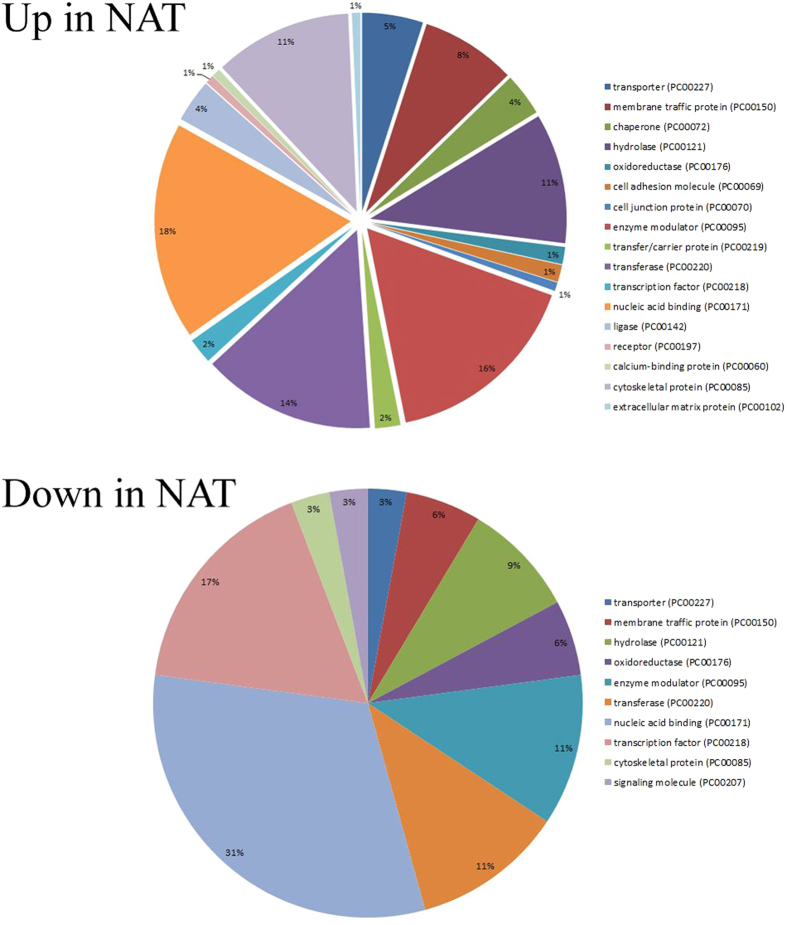
Functional categories of differentially regulated genes in NAT. Differentially regulated genes in NAT were classified according to the protein class that they encode using the online PANTHER Version 11.0 (http://pantherdb.org/). The settings for the PANTHER analysis are described in the methods section. The percentage showed in the pies corresponds to the percent of gene hit against total # Protein Class hits setting.

We compared the gene expression profiles of NAT and control trophozoites using RNA deep sequencing. When a p < 0.01 was set as the cutoff value, we found 208 genes were upregulated and 124 genes were downregulated in NAT (Supporting Information S1). The differentially regulated genes in NAT and the control trophozoites were classified according to the protein class which they encode (Fig. 2) using the Protein ANalysis THrough Evolutionary Relationship (PANTHER) sequence classification tool^18^,^19^. The upregulated genes in NAT included those which encode chaperone proteins, such as HSP710 (EHI_130160) and DnaJ domain-containing proteins (EHI_170090), cytoskeletal proteins, such as actin (EHI_126190) and filopodin (EHI_167130), proteins associated with signal transduction, such as RAP/RAN GTPase activating protein (EHI_079910) and serine/threonine kinase 3 (EHI_096560), hydrolases, such as 26S protease regulatory subunit (EHI_052050), CP 17 (EHI_080890) and acetylornithine deacetylase (EHI_114340), membrane vesicle trafficking proteins, such as adaptor proteins (EHI_023600 and EHI_058450), and nucleic acid binding proteins, such as 40S ribosomal protein S4 (EHI_057850) and translation initiation factor (EHI_189410)). Of the upregulated genes in NAT, genes that are associated with actin family cytoskeletal protein such as actin (EHI_126190), filopodin (EHI_167130), actinin (EHI_155290), WD repeat (EHI_118160), alpha actinin (EHI_146140) and coronin (EHI_105330) are significantly enriched according to the PANTHER statistical overrepresentation test. Of the downregulated genes, no enrichment of a specific biological pathway was detected according to the PANTHER statistical overrepresentation test.

### NAOD is involved in the adaptation of *E. histolytica* to NS

**Figure 3:**
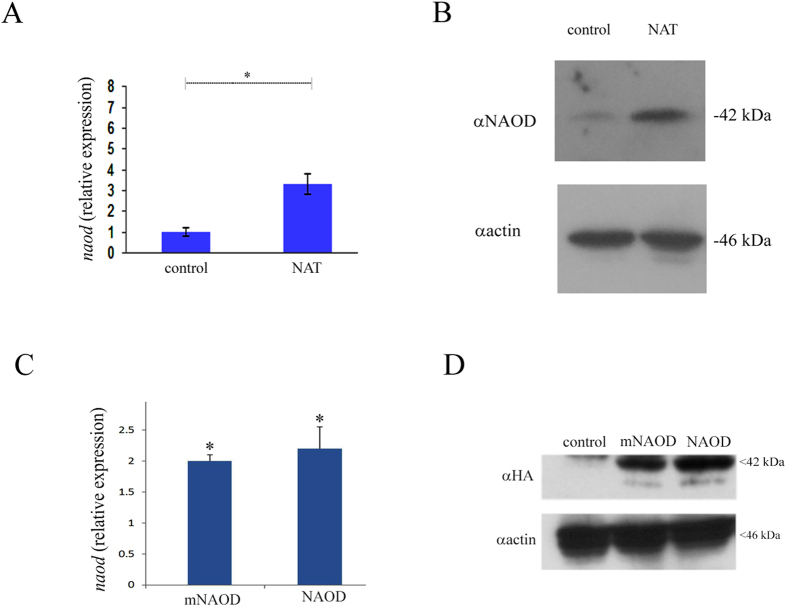
NAOD expression in NAT, NAOD trophozoites and mNAOD trophozoites. (**A**) qRT-PCR was used to monitor *naod* expression using cDNA from NAT. The QRT-PCR values were normalized to the level of rDNA^74^. Data are expressed as the mean ± SD of three independent experiments. *p ≤ 0.05 and is the significance of the difference between the expression levels of *naod* in the control trophozoites and NAT according to the results of an unpaired Student’s t-test. (**B**) Western blot analysis was performed on total protein extracts that were prepared from control trophozoites and NAT. The proteins were separated on 12% SDS-PAGE gels and analyzed by western blotting with a NAOD antibody or actin antibody. The figure displays a representative result from three independent experiments. (**C**) qRT-PCR was used to monitor *naod* expression using cDNA from pcontrol trophozoites, NAOD trophozoites and mNAOD trophozoites. The QRT-PCR values were normalized to the level of *naod* expression in pcontrol trophozoites. Data are expressed as the mean ± SD of three independent experiments. *p ≤ 0.05 and is the significance of the difference between the expression levels of NAOD in the pcontrol trophozoites, NAOD trophozoites and mNAOD trophozoites according to the results of an unpaired Student’s t-test. (**D**) Western blot analysis was performed on total protein extracts that were prepared from pcontrol trophozoites, NAOD trophozoites and mNAOD trophozoites. The proteins were separated on 12% SDS-PAGE gels and analyzed by western blotting with an HA antibody or actin antibody. Cropped image of representative results from three independent experiments. Full length image is presented in Supporting Information S4.

**Figure 4:**
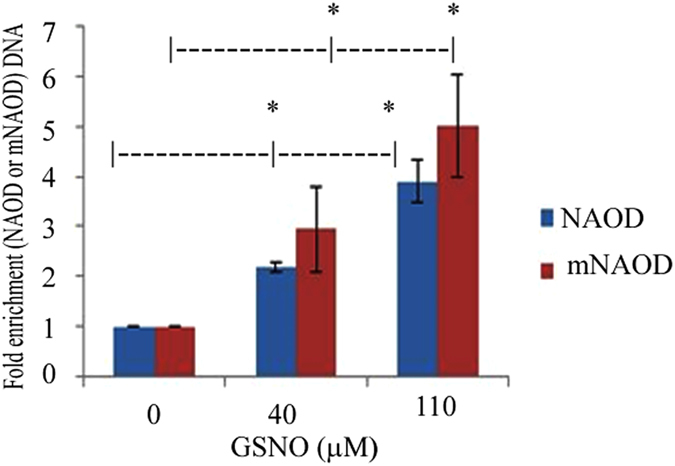
Overexpression of NAOD or overexpression of mNAOD provides selective advantage during adaptation to NO. (**A**) Competition between pcontrol trophozoites and NAOD trophozoites or pcontrol trophozoites and mNAOD trophozoites during adaptation to NO. qPCR analysis of *NAOD* or m*NAOD* and *neo* was performed on extrachromosomal DNA that was prepared from mix cultures that have been adapted to 40 and 110 μM GSNO. The level of *neo* which is carried by pcontrol plasmid, NAOD plasmid and mNAOD plasmid was used to normalize the data. Data are expressed as the mean ± SD of three independent experiments. *p ≤ 0.05 and is the significance of the difference between the amount of NAOD DNA or mNAOD DNA in the trophozoite population cultivated in absence of GSNO or in presence of 40 or 110 μM GSNO according to the results of an unpaired Student’s t-test.

**Figure 5:**
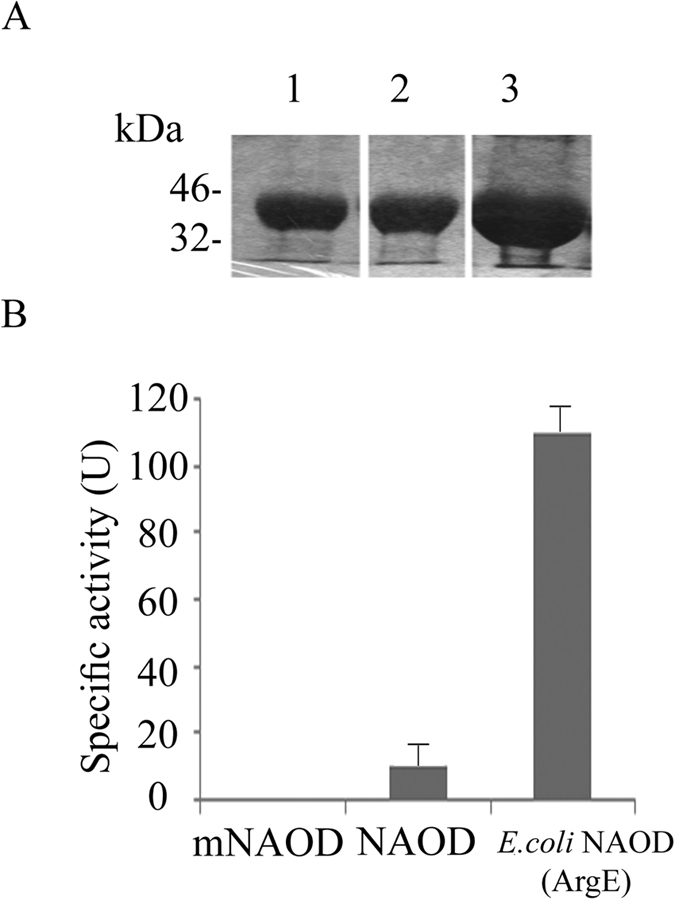
Purification of His-tagged *E. histolytica* NAOD, His-tagged *E. histolytica* mNAOD and His-tagged *E. coli* NAOD (ArgE). (**A**) Analysis of eluted His-tagged NAOD, His-tagged mNAOD and His-tagged His-tagged *E. coli* NAOD (ArgE) was determined by SDS-PAGE and Coomassie staining (A cropped image is showed. Full length blots are presented in Supporting Information S4). Lane 1, His-tagged mNAOD, Lane 2 His-tagged NAOD, Lane 3 His-tagged *E. coli* NAOD (ArgE). (**B**) Specific activity of His-tagged mNAOD, His-tagged NAOD, and His-tagged *E. coli* NAOD (ArgE). One unit (U) corresponds to 1 nmol ornithine produced/in/mg protein.

We have recently showed that the accumulation of the polyamine putrescine and *E. histolytica*’s resistance to OS are linked^20^. The synthesis of polyamines has been also linked to NS resistance in bacteria^21^,^22^. This information encouraged us to seek individual gene(s) which are involved in polyamine synthesis and in the adaptation of the parasite to NO. NAOD (EHI_114340) was chosen as a candidate gene because NAOD expression is significantly increased in NAT (~2.5 times) (Supporting Information S1, Fig. 3A,B). In addition, NAOD catalyzes the deacylation of N^2^-acetyl-L-ornithine to yield ornithine and acetate. Ornithine is an obligatory intermediate in the arginine biosynthetic pathway and a branch point in the synthesis of polyamines. Polyamines are known for their protective properties against NS and OS^23^,^24^,^25^. In addition, NAOD expression is upregulated in *E. coli* which are exposed to peroxynitrite^26^. To test the hypothesis that NAOD plays a role in the adaptation of the parasite to NS, we first tried to downregulate NAOD expression using the antisense approach^27^ and the G3-based approach^28^ without success which suggest that NAOD is essential for the parasite (data not shown). We then tried to upregulate NAOD expression in *E. histolytica* trophozoites HMI:IMSS strain (NAOD trophozoites) as a hemagglutinin (HA)-tagged protein. Overexpression of NAOD mimics the upregulation of this gene in trophozoites that were exposed to an acute NS and in NAT. Overexpression of NAOD was confirmed by quantitative reverse transcription PCR (RT-qPCR) and by immunoblot analysis using HA antibody (Fig. 3C,D). We first determined whether NAOD confers a selective advantage for the adaptation to increasing amount of GSNO. For this purpose, pcontrol and NAOD trophozoites that were cultivated in 6 μg/ml G418 were mixed (1:1) and incubated in TYI-33 medium without G418. This mixed population of trophozoites was then exposed to increasing amounts of GSNO up to 110 μM. Extrachromosomal DNA was prepared from trophozoites that were adapted to 40 and 110 μM GSNO and the level of NAOD DNA in these culture was measured by qPCR and normalized to the level of neo gene which is carried by the control vector and by the NAOD vector. We found that exposure of pcontrol and NAOD trophozoites to increasing amounts of GSNO resulted in the enrichment of NAOD trophozoites in the population (Fig. 4). This result prompted us to further investigate why NAOD confers a selective advantage to *E. histolytica* trophozoites that were exposed to NS. NAOD catalyzes the deacylation of N^2^-acetyl-L-ornithine to yield ornithine and acetate. We posited that this selective advantage is conferred by NAOD’s enzymatic activity. We found that *E. histolytica* NAOD is a functional enzyme even if its specific activity is ten times less than that of recombinant *E. coli* NAOD (ArgE) (Fig. 5A,B). We also found that the intracellular putrescine and acetate concentrations in NAOD trophozoites (303 ± 25 μM) and (1075 ± 45 μM), respectively were significantly higher than those in the pcontrol trophozoites (119 ± 15 μM) and (350 ± 30 μM). No significant difference in the intracellular ornithine concentration in pcontrol (3.4 ± 0.2 μM) and NAOD trophozoites (3.0 ± 0.2 μM) was detected. These results suggest that the protective effect of NAOD against NS is related to the elevated intracellular concentration of putrescine in NAOD trophozoites. Ornithine, the product of NAOD reaction, is converted into putrescine by ornithine decarboxylase (ODC)^29^,^30^. Putrescine is free radical scavenger and an antioxidant^31^. To test the hypothesis that the protective effect of NAOD against NS is the result of high levels of putrescine in the NAOD trophozoites, NAOD protein with one of its zinc binding sites deleted (DMKG; amino acid 108 to 113) (mNAOD) was overexpressed in *E. histolytica* (Fig. 3C,D). The ability of mNAOD trophozoites and control trophozoites to adapt to increasing amounts of GSNO was compared (Fig. 4). To our surprise, we found that mNAOD trophozoites better adapt to GSNO than pcontrol trophozoites (Fig. 4) despite the fact that mNAOD is catalytically inactive (Fig. 5B). We also found that the intracellular putrescine and acetate concentrations in mNAOD trophozoites (135 ± 20 μM) and (275 ± 25 μM) were comparable to those in pcontrol trophozoites. These results indicate that the catalytic activity of NAOD and high intracellular concentrations of putrescine are not essential for the protective effect that NAOD confers against NS.

**Figure 6:**
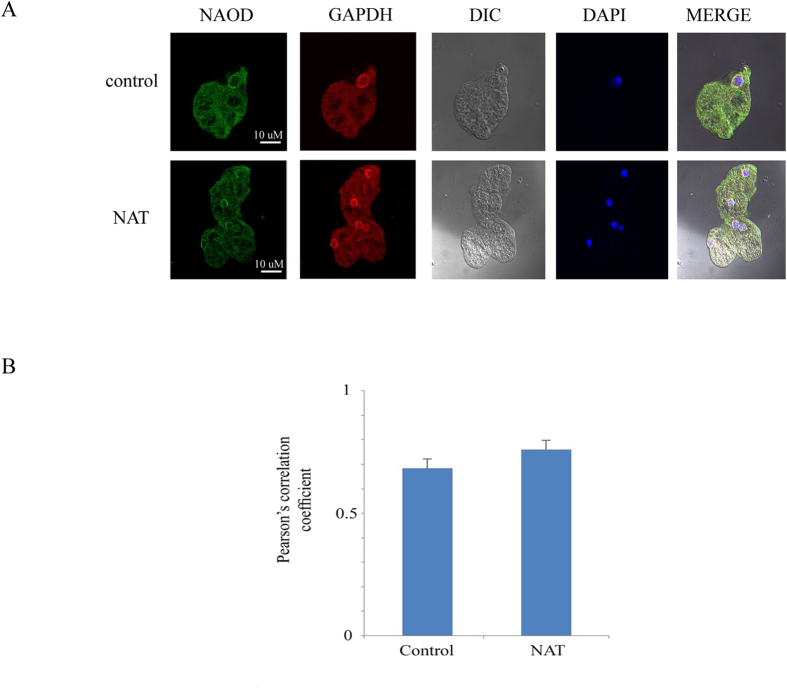
Confocal laser scanning microscopy of NAOD and GAPDH in control trophozoites and NAT. (**A**) NAOD was detected using primary NAOD antibody and secondary Cy2-conjugated IgG antibody. GAPDH was detected using primary GAPDH antibody and secondary Cy3-conjugated IgG antibody. The nuclei (blue) were stained by DAPI. Computer-assisted image overlay of the signal emitted by the NAOD antibody, the GAPDH antibody and DAPI revealed showed that NAOD and GAPDH are located in the cytoplasm and perinuclear region of control trophozoites and NAT. (**B**) Analysis of Pearson’s correlation coefficient of colocalization between NAOD and GAPDH in the perinuclear area. The analysis has been performed on eight independent images for control trophozoites and six independent images for NAT. Data are expressed as the mean ± SD.

In order to further understand the function of NAOD, we determined its intracellular location using an NAOD antibody and immunofluorescence confocal microscopy (Fig. 6). Computer-assisted image overlay of the signal that was emitted by the NAOD antibody and DAPI, a specific nuclear stain, revealed that NAOD is located in the cytoplasm and the perinuclear region of pcontrol trophozoites and NAT (Fig. 6A).

### NAOD interacts with GAPDH

**Figure 7:**
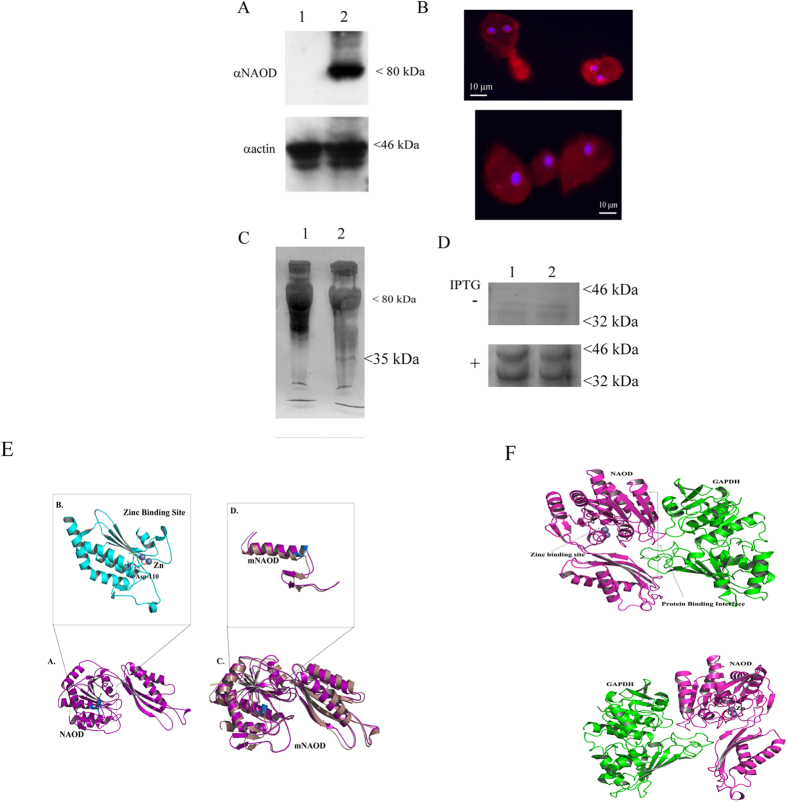
NAOD and GAPDH interact. (**A**) Western blot analysis of whole protein extract prepared from HaloTag control trophozoites (lane 1) and from HaloTag-NAOD trophozoites (lane 2). The proteins were separated on 10% SDS-PAGE gels and analyzed with an NAOD antibody (αNAOD) or an actin antibody (αactin). The cropped image displays a representative result from three independent experiments. Full length blot is presented in Supporting Information S4. (**B**) Immunofluorescence confocal microscopy of HaloTag-NAOD trophozoites. HaloTag-NAOD (red) was detected using HaloTag TMR Ligand (1 μM). The nuclei (blue) were stained by DAPI. No fluorescence was observed when Halo-tagged trophozoites were incubated with HaloTag TMR Ligand. (**C**) HaloCHIP of NAOD from HaloTag-NAOD trophozoites exposed of not to GSNO (350 μM for 30 min). A 35 kDa protein is specifically pull-down from the whole protein extract of HaloTag-NAOD trophozoites. (**D**) NAOD (or mutNAOD) and GAPDH are co-expressed from the pETDuet-1 expression vector in *E. coli*. In this vector, NAOD (lane 1) or mNAOD (lane 2) was expressed as a His-tagged protein and GAPDH was untagged. His-tagged NAOD or mNAOD was pull-downed from non-IPTG-induced *E. coli* or IPTG-induced *E. coli* lysates with Ni-NTA resin. The pull-downed proteins were resolved on an SDS-PAGE gel and the gels were Coomassie stained. (**E**) Model of NAOD (purple color). (**A**) The structure shows the binding of Zinc at the active site along with interacting residues. (**B)** Zoomed view of the zinc binding site (cyan color) along with the ASP-110 in stick model. (**C)** Superimposition of mNAOD (violet color) with full length NAOD. (**D)** Zoomed view of the zinc binding site in mNAOD structure compared to the full length model. (**F**) Model of NAOD-GAPDH interaction. (upper panel). Final complex (best pose) obtained from the docking simulation of NAOD-GAPDH interaction. The figure shows the binding site of Zinc and the protein-protein interaction site between NAOD and GAPDH. (lower panel). 180 degree rotated view of the interaction between NAOD and GAPDH.

Many biological processes, such as adaptation and resistance of organisms to NS, are contingent on protein-protein interactions^32^. Accordingly, we conducted a series of experiments whose aims were to identify those proteins which interacted with NAOD in order to understand its function in the mechanism(s) of adaptation and resistance of *E. histolytica* trophozoites to NS. To this end, we successfully detected NAOD and its interacting proteins using the HaloTag mammalian pull-down and labeling systems. The expression of NAOD HaloTag fusion protein in *E. histolytica* trophozoites (HaloTag-NAOD trophozoites) but not in the HaloTag control trophozoites was confirmed using western blot analysis (Fig. 7A) and confocal fluorescence microscopy (Fig. 7B). We also observed that HaloTag-NAOD trophozoites have a level of resistance to NS that was comparable to that of HA-tagged-NAOD trophozoites (data not shown).

We recovered using HaloChIP a 35 kDa protein that was bound to the NAOD HaloTag fusion protein. This 35 kDa protein was captured from the lysates of nitrosatively-stressed HaloTag-NAOD trophozoites (Fig. 7C). This 35 kDa protein was identified as glyceraldehyde 3-phosphate dehydrogenase (GAPDH) by mass spectrometry (Supporting Information S2). The binding of NAOD to GAPDH was confirmed by co-expression in *E. coli* of His-tagged NAOD (the expression of His-tagged NAOD was induced by IPTG) and untagged-GAPDH, both of which are expressed by the pETDuet-1 vector, and their co-purification with nickel beads that were used to pull-down His-tagged NAOD (Fig. 7D). The nature of His-tagged NAOD and GAPDH after their co-purification was confirmed by mass spectrometry (Supporting Information S3). We also found that His-tagged mNAOD was efficiently expressed in *E. coli* from the pETDuet-1 vector and that His-tagged mNAOD binds to GAPDH (Fig. 7D). As a negative control, the co-purification was performed from non-induced *E. coli* cells (-IPTG) carrying the pETDuet-1 His-tagged NAOD- untagged-GAPDH vector or the pETDuet-1 His-tagged mNAOD- untagged-GAPDH (Fig. 7D). No NAOD (or mNAOD) expression was detected in the non-induced *E. coli* cells resulting in the absence of GAPDH co-purification (Fig. 7D).

In order to characterize the molecular basis of the NAOD-GAPDH interaction, structural models of NAOD and mNAOD were first built using the crystal structure of zinc-bound acetylornithine deacetylase from *Rhodopseudomonas palustris.* The final energy-minimized structure (Fig. 7E) of the model with zinc at the active site strongly indicate that the stretch of residues (DMKG; amino acids 110 to 113) is crucial for holding zinc at the reaction center (active site). The binding of zinc (Fig. 7E) in the acetylornithine deacetylase structure from Rhodopseudomonas palustris suggested that the negatively charged ASP-110 as well as MET-111 are directly involved in the binding. The structural model of mNAOD devoid of the zinc binding residues, when superimposed over the full length NAOD has a root mean square deviation of 1.866 (327 to 327 atoms). The differences at the active site are shown in Fig. 7E. The mNAOD model clearly indicates that the deletion the stretch of residues (DMKG; amino acids 110 to 113) causes the disruption of the zinc binding domain.

*E. histolytica* GAPDH was modeled using the crystal structure of *Leishmania mexicana* GAPDH^33^, and protein-protein docking simulations (Cluspro and Z-dock) were used to predict the interactions between NAOD and GAPDH. The results of these simulations indicated that GAPDH binds to the negatively charged interface of NAOD which is located far from the zinc binding domain (Fig. 7F). The α-7 helix in the NAOD structure is the master key of the NAOD and GAPDH interaction. This model explains why the binding of NAOD to GAPDH does not inhibit GAPDH activity. In addition, the α-7 helix and the negatively charged interface of NAOD are still functional in mNAOD (Fig. 7E) which explain why mNAOD can also bind GAPDH.

In order to further characterize the NAOD-GAPDH interaction, we investigated whether GAPDH co-localizes with NAOD in control trophozoites and NAT using immunofluorescence confocal microscopy. Computer-assisted image overlay of the signal emitted by the GAPDH antibody, the NAOD antibody, and DAPI and Pearson’s correlation coefficient revealed that GAPDH and NAOD co-localize (Fig. 6A,B). Specifically, GAPDH and NAOD co-localize mainly in the perinuclear region in the control trophozoites and NAT (Fig. 6A,B).

GAPDH catalyzes the conversion of glyceraldehyde-3-phosphate to 1,3-bisphosphoglycerate. The binding of NAOD to GAPDH raises a question about the effect of this interaction on GAPDH activity. In order to answer this question, GAPDH activity in the total protein extract of *E. histolytica* trophozoites was determined before and after pre-incubation of the extract with increasing amounts of NAOD. GAPD activity in the protein extract of *E. histolytica* trophozoites (2.15 nanomoles of NADH produced/min/mg) was not affected by increasing amounts of NAOD. GAPDH activity was also determined in the total protein extracts of HaloTag control trophozoites and HaloTag-NAOD trophozoites. GAPDH activity was found to be equivalent in these total protein extracts (data not shown). Collectively, these results suggest that NAOD has no effect on GAPDH activity.

**Figure 8:**
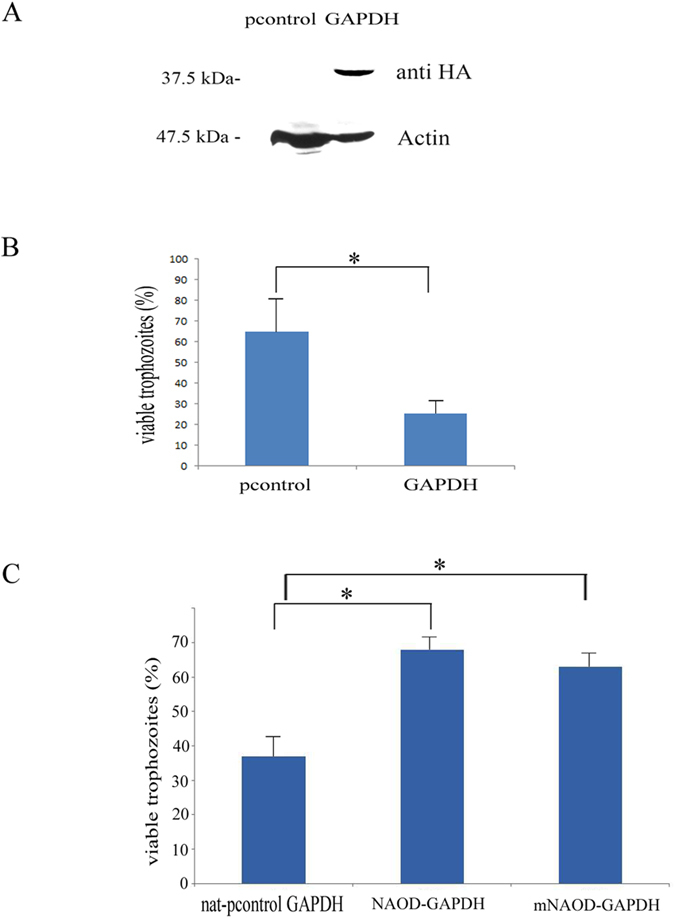
The toxic effect of GAPDH on TEANS is neutralized by NAOD and by mNAOD. (**A**) Western blot analysis was performed on total protein extracts that were prepared from pcontrol trophozoites and GAPDH trophozoites. The proteins were separated on 12% SDS-PAGE gels and analyzed by western blotting with an HA antibody or actin antibody. The figure displays a representative result from three independent experiments. (**B**) Viability of pcontrol trophozoites and GAPDH trophozoites that were exposed to 350 μM GSNO for 60 minutes. The number of trophozoites at the beginning of each experiment was set at 100%. Data are expressed as the mean and SD of three independent experiments that were done in triplicate. The viability of GAPDH trophozoites was significantly different (p < 0.05) from that of the pcontrol trophozoites according to the results of an unpaired Student’s t-test. (**C**) Viability of nat-pcontrol GAPDH trophozoites, NAOD-GAPDH trophozoites and mNAOD-GAPDH trophozoites that were exposed to 350 μM GSNO for 60 minutes. The number of trophozoites at the beginning of each experiment was set at 100%. Data are expressed as the mean and SD of three independent experiments that were done in triplicate. The viability of NAOD-GAPDH trophozoites and mNAOD-GAPDH trophozoites was significantly different (p < 0.05) from that of the nat-pcontrol GAPDH trophozoites according to the results of an unpaired Student’s t-test.

Recent studies have shown that GAPDH has multiple functions independent of its role in energy metabolism (for a recent review see ref. 34). GAPDH expression is increased in *Leishmania* spp. that are naturally resistant to NS^35^ which suggest that GAPDH has a protective role against NS in this parasite. In contrast GAPDH can trigger cell death in oxidatively- and nitrosatively-stressed mammalian cells^36^,^37^,^38^. GAPDH expression is upregulated in trophozoites exposed to NS^5^ however we have no knowledge about the beneficial or detrimental effect that GAPDH has on *E. histolytica* exposed to NS. In order to fill this knowledge gap, GAPDH was overexpressed in *E. histolytica* trophozoites HMI:IMSS strain (GAPDH trophozoites) as a (HA)-tagged protein. Overexpression of GAPDH mimics the upregulation of this gene in trophozoites that were exposed to an acute NS^5^. Overexpression of GAPDH was confirmed by immunoblot analysis using HA antibody (Fig. 8A). Pcontrol trophozoites and GAPDH trophozoites were exposed to GSNO (350 μM) for 60 minutes (Fig. 8B). We observed that GAPDH trophozoites are more sensitive to NS than pcontrol trophozoites (Fig. 8B). In order to determine if the detrimental effect of GAPDH in trophozoites exposed to NS is reduced by NAOD or by mNAOD, we overexpressed NAOD or mNAOD as (HA)-tagged proteins in GAPDH trophozoites (NAOD-GAPDH and mNAOD-GAPDH trophozoites). HA-NAOD or HA-mNAOD were carried by a modified pcontrol vector^39^ were the neo gene that confers resistance to G418 has been replaced by the nat gene that confers resistance to nourseothricin (nat-pcontrol). The presence of the respective vectors in nat-pcontrol GAPDH trophozoites, NAOD-GAPDH trophozoites and mNAOD-GAPDH trophozoites was confirmed by plasmid rescue into *E. coli* TOP10. We observed that NAOD-GAPDH and mNAOD-GAPDH trophozoites are more resistant to NS than nat-pcontrol GAPDH trophozoites (Fig. 8C). Collectively, these results strongly suggest that the protective effect of NAOD against NS is related to the reduction of GAPDH detrimental effect in trophozoites exposed to NS.

## Discussion

In order to survive and grow in its human host, *E. histolytica* must monitor changes in its environment and respond accordingly by adjusting the expression of its stress- and virulence-associated genes. We and others have previously reported on the parasite’s ability to adapt to various stresses, such as glucose starvation^11^, serum starvation^40^ or treatment with metronidazole^41^. In this report, we inform on the ability of *E. histolytica* to adapt to NS. Adaptation to NS has been documented in some microorganisms, such as *Bacillus subtilis*^42^ and *Leishmania donovani*. In *L. donovani*, redox proteins, such as thioredoxin and peroxidoxin, are essential for the parasite’s adaptation to NS^43^. The expression of redox proteins is also upregulated in acute nitrosative-stressed trophozoites^5^. Despite the fluctuations in the regulation of expression of genes which encode various classes of proteins, we did not find enrichment of redox proteins in NAT. In fact, NAT seem to have reached a state of homeostatic equilibrium after their adaptation to NS. This state of homeostatic equilibrium is characterized by the differential expression of 332 genes and an increased resistance to activated macrophages.

Of the upregulated genes in NAT, genes that are associated with actin family cytoskeletal protein are significantly enriched. *E. histolytica* relies on its dynamic actin cytoskeleton to migrate within various compartments of the human body (for a recent review see ref. 44). According to previous reports in mammalian cells, the cysteines in actin are some of the most susceptible targets of oxidation and nitrosylation and their modification is the likely cause of reduced polymerization activity and altered binding of actin with actin binding proteins^45^. We have previously reported that a number of cysteine residues in *E. histolytica* actin are susceptible to oxidation in oxidatively stressed trophozoites^20^ and to S-nitrosylation in NAT (manuscript in preparation). Based on this information, it is tempting to speculate that the upregulation of cytoskeletal proteins in NAT is a mechanism used by the parasite to replace SNO cytoskeletal proteins.

We found that GalNAc lectin in NAT is functional and that GalNAc lectin binding activity is inhibited in TEANS^15^. We also found that GalNAc lectin in NAT is not S-nitrosylated (data not shown), and this finding suggests that S-nitrosylation of GalNAc lectin did not occur, was blocked, or even reversed. The 29-kDa thiol-dependent peroxidase, Eh29, may be involved in preventing S-nitrosylation of GalNAc lectin because Eh29 binds the GalNAc lectin and it is involved in the survival of *E. histolytica* against ROS^46^,^47^. GAPDH is another good candidate because it interacts with the GalNAc lectin^48^. A third candidate that we are considering for further analysis is thioredoxin which can restore the activity of *E. histolytica* serine acetyltransferase-1 that was impaired by oxidation^49^ and restore the damaging effect of S-nitrosylation on *Helicobacter pylori* arginase activity^50^.

Adaptation of microorganisms to stress is often associated with a fitness cost. However, this fitness cost can be negligible for an adapted microorganism as long as the selection pressure or the stress is maintained^51^. NAT have a lower growth rate and an increased sensitivity to OS and metronidazole than control trophozoites. The growth rate of metronidazole-adapted *E. histolytica* trophozoites and NAT is low but metronidazole-adapted *E. histolytica* trophozoites are more resistant to OS than NAT^52^. Iron-sulfur flavoproteins are among the most upregulated genes in metronidazole-adapted *E. histolytica* trophozoites^52^ and L-cysteine starved and oxidatively-stressed trophozoites^53^,^54^. It has also been reported that the expression of iron-sulfur flavoproteins is upregulated in TEANS^5^. Such findings suggest that upregulating the expression of iron-sulfur flavoproteins expression is an integral part of the parasite’s response to an environmental stress. In addition, the repression of iron-sulfur flavoproteins expression in *E. histolytica* is associated with a reduced growth rate^54^. Surprisingly, three iron-sulfur flavoproteins (EHI_103260, EHI_134740, EHI_067720) were among the most downregulated genes in NAT. The low level of expression of iron-sulfur flavoproteins in NAT could account for the low generation time and the increased sensitivity to OS and metronidazole of NAT. These data also suggests that a high level of iron-sulfur flavoproteins expression is detrimental to NAT but the reason for this detrimental effect still requires elucidation.

We found that NAOD expression is upregulated in NAT. NAOD (ArgE) expression in *E. coli* is upregulated when the bacteria are exposed to peroxynitrite^26^ but the reason for this upregulation is unknown. We also found that NAOD is involved in the parasite’s response to NS. Surprisingly, this function does not rely on catalytic activity of NAOD and on the formation of putrescine. These results suggest that this protein is a moonlighting protein. We have previously showed that the glycolytic enzyme, enolase, in the parasite is a moonlighting protein which regulates the activity of Ehmeth^55^. However, examples of moonlighting proteins which are directly involved in the resistance to reactive oxygen or nitrogen species or are regulated by reactive oxygen or nitrogen species are rare. One example is the involvement of the cell wall proteins of *Candida albicans* in protecting the fungus against OS^56^. Another example is GAPDH that triggers cell death following its S-nitrosylation^57^. We have previously reported that S-nitrosylated NAOD has not been detected in TEANS^15^ which suggests that NAOD is not regulated by S-nitrosylation. A clue about the function of NAOD in the parasite’s ability to adapt to NS may come from those experiments which investigated its binding to GAPDH. GAPDH interacts with numerous proteins of diverse functions (for a review see ref. 58). To the best our knowledge, this is the first report which describes an interaction between GAPDH and NAOD. GAPDH interacts with many cellular proteins and the outcome of each interaction is different. For example, the interaction between GAPDH and SIAH1 results in a change in the intracellular location of GAPDH^59^. The interaction between GAPDH and FKBP36 results in the inhibition of GAPDH activity^60^. In contrast, the interaction between GAPDH and “p300/(CREB binding protein) Associated Factor” (PCAF) results in the activation of GAPDH activity^61^. We found that the activity of GAPDH does not change when GAPDH binds to NAOD. This result is supported by our model of the interaction between NAOD and GAPDH: formation of an NAOD-GAPDH complex does not involve GAPDH’s active site.

On the one hand, upregulation of GAPDH expression is correlated with resistance to NS in *Leishmania* spp.^35^ and it has been proposed that GAPDH may act as a NO scavenger^62^. On the other hand the formation of toxic GAPDH species in oxidatively- and nitrosatively-stressed cells is involved in the pathogenesis of Alzheimer’s disease^38^ and Huntington’s disease^36^. Our findings support a detrimental role of GAPDH in trophozoites exposed to NS. Although we will need further investigation to understand the exact mechanism of GAPDH toxicity in *E. histolytica* exposed to NS, our findings clearly indicate that NAOD acts by neutralizing the toxic effect of GAPDH. Strategies that counteract the protective effect of NAOD may be valuable in the struggle against this parasite.

To conclude, we have described the phenotype of NO-adapted *E. histolytica*. Although it is difficult to deduce from our data whether such adaptation to NO actually occurs when the parasite resides in its host, this investigation highlights the parasite’s amazing ability to adapt to various stress conditions^10^,^52^. We have previously reported that enolase is a moonlighting protein in *E. histolytica*^55^. This investigation revealed that NAOD is also a moonlighting protein in *E. histolytica* and it highlights the contribution of moonlighting proteins to the biology of parasitic protozoa^63^.

## Methods

### Chemicals and Reagents

*S*-nitrosoglutathione (GSNO), metronidazole, H_2_O_2_, and G418 were purchased from Sigma-Aldrich, St. Louis, MO, USA. A stock solution of S-nitroso-L-cysteine (CysNO) was prepared according to a previously reported method^64^ in which 0.2 M NaNO_2_ is mixed with 0.2 M cysteine in HCl, and the mixture is then neutralized using NaOH [30]. The concentration of CysNO was determined spectrophotometrically using an extinction coefficient of 900 M^−1^cm^−1^. Under these conditions, the yield of CysNO was more than 90%.

### Microorganisms

*E. histolytica* trophozoites strain HM-1:IMSS^65^ (a gift of Prof. Mirelman, Weizmann Institute) were grown under axenic conditions in Diamond’s TYI-S-33 medium^66^ at 37 °C. Trophozoites in the exponential phase of growth were used in all experiments.

*Escherichia coli* strain TOP1O (Invitrogen) was systematically used for cloning.

*E. coli* strain BL21(DE3)pLysS (Promega) was used for protein expression.

### Adaptation to GSNO

Trophozoites which were able to grow in medium that contained 120 μM GSNO were produced from trophozoites that were initially grown for one week in medium that contained 30 μM GSNO and then grown in medium which contained stepwise increases in the GSNO concentration. These GSNO-adapted trophozoites, which were produced after four weeks, were used in all experiments. The generation time of GSNO-adapted and control HM-1:IMSS trophozoites is defined as the time (in hours)/number of generations.

### Cultivation of HeLa Cells and RAW 264.7 murine macrophage cell

HeLa cells (Sigma, The Health Protection Agency (HPA) Culture Collections) and the RAW 264.7 murine macrophage cell line (Sigma, HPA Culture Collections) were maintained in continuous culture in 100-mm plates in Dulbecco’s modified Eagle’s medium (DMEM) (Biological Industries, Kibbutz Beth HaEmek, Israel) which was supplemented with 2 mM glutamine, 100 units/ml penicillin, 100 μg/ml streptomycin, and 10% (v/v) fetal calf serum at 37 °C under a humidified 5% CO_2_ atmosphere.

### DNA constructs

For construction of the pJST4-NAOD vector, NAOD was amplified from *E. histolytica* cDNA using the primers, NAOD KpnI and NAOD BamH1 (Table 1). The PCR product was subcloned using the pGEM-T Easy vector system (Promega) and then digested with the restriction enzymes, KpnI and BamHI. The digested DNA insert was cloned into the *E. histolytica* expression vector pJST4 which had been previously linearized with KpnI and BglII. The pJST4 expression vector (pcontrol) contains a tandem affinity purification tag for use in protein purification and identification^39^. This CHH-tag contains the calmodulin binding protein, hemagglutinin (HA), and histidine (His) residues and its expression is driven by an actin promoter. This vector was used as the control in our experiments in order to exclude the possibility that the CHH-tag is responsible for the phenotypes of the NAOD-overexpressing strain.

For the construction of the pJST4-mNAOD, a synthetic NAOD gene with deletion of nucleotides 327 to 338 (mNAOD) (Syntezza Bioscience Ltd) was cloned into the pJST4 vector in the same manner that was used for cloning the non-mutated NAOD gene.

For the construction of the nat-pJST4-NAOD vector, nat gene was amplified from the KB2251 plasmid (a kind gift from Kornitzer D., Faculty of Medicine, Technion) using nat5′KpnI and nat3′BamHi primers. Nat was cloned into the vector pJST4 which had been previously linearized with KpnI and BamHI. Nat bordered by *E. histolytica* actin 5′ and actin 3′ regions was amplified from the previous construction using actin 5′XhoI and actin 3′XhoI primers. The PCR product was subcloned using the pGEM-T Easy vector system (pGEM-T easy nat) and then digested with the restriction enzymes, XhoI. The digested DNA insert was cloned into the vector pJST4-NAOD which had been previously linearized with XhoI. This procedure led to the replacement of the neo gene in the pJST4-NAOD vector by the nat gene to produce the nat-pJST4-NAOD vector.

For the construction of the nat-pJST4-mNAOD vector, pJST4-mNAOD was linearized with XhoI and the neo gene was replaced by the nat gene from the pGEM-T easy nat vector that was previously linearized with XhoI. For the construction of the nat-pJST4 vector (nat-pcontrol), pJST4-NAOD was linearized with KpnI and BamHI to remove NAOD, end blunt with T4 DNA polymerase and self-ligated. The resulting vector was linearized with XhoI and the neo gene was replaced by the nat gene from the pGEM-T easy nat vector that was previously linearized with XhoI.

For the construction of Halo-tagged NAOD expression vector, NAOD was amplified from *E. histolytica* cDNA using sense and antisense primers with a BamHI site (Table 1). The PCR product was then cloned in the HaloTag expression vector (a kind gift from T. Nozaki, National Institute of Infectious Diseases, Tokyo, Japan) that was previously linearized with Bgl II. The HaloTag expression vector allows the expression of a Halo-tagged protein in *E. histolytica* that is driven by a cysteine synthase promotor.

These constructs were transfected into *E. histolytica* trophozoites using a previously described protocol^67^. The transfected trophozoites were subcultured continuously in the presence of 6 μg /ml or 40 μg/ml G418 (when the resistance to G418 (Sigma-Aldrich) which is conferred by *neo* is present in the construct) or in the presence of 6 μg/ml or 40 μg/ml nourseothricin (Sigma-Aldrich) (when the resistance to nourseothricin which is conferred by *nat* is present in the construct). Plasmid DNA rescue from pcontrol trophozoites, NAOD-, and mNAOD-overexpressing trophozoites in *E. coli* was performed using a previously described protocol^68^.

For construction of the pET28b NAOD vector, NAOD was amplified from *E. histolytica* cDNA by PCR using the primers, NcoI NAOD and XhoI NAOD (Table 1). The PCR product was then cloned in a pGEM-T easy vector, digested with NcoI and XhoI, and then subcloned into the pET28b vector that was previously linearized with NcoI and XhoI.

For construction of the pET28b mNAOD vector, the synthetic mNAOD gene was amplified with the primers, NcoI NAOD and XhoI NAOD (Table 1). The PCR product was then cloned in a pGEM-T easy vector, digested with NcoI and XhoI, and then subcloned into the pET28b vector that was previously linearized with NcoI and XhoI.

For construction of the pET28b, ArgE vector, ArgE was amplified from *E. coli* gDNA with the primers, ArgE NcoI and ArgE XhoI (Table 1). The PCR product was then cloned in a pGEM-T easy vector, digested with NcoI and XhoI, and then subcloned into the pET28b vector that was previously linearized with NcoI and XhoI.

For construction of the pETDuet-1 His-tagged NAOD-GAPDH vector, NAOD was amplified from *E. histolytica* cDNA with the primers BamHI NAOD and antisense NAOD (Table 1). The PCR product was cloned in a pGEM-T easy vector, digested with BamHI and NotI, and then subcloned in a pDuet expression vector that was previously linearized with BamHI and NotI to generate the pETDuet-1 His-tagged NAOD vector. GAPDH was then amplified from *E. histolytica* cDNA by using primers EcoRV GAPDH and KpnI GAPDH primers, and then subloned in the pETDuet-1 His-tagged NAOD vector that was previously linearized with EcoRV and KpnI. For construction of the pETDuet-1 His-tagged mNAOD-GAPDH vector, the same cloning strategy described above was used except that mNAOD was used as DNA template in the first amplification with the primers BamHI NAOD and antisense NAOD (Table 1).

All constructs described in this section were verified by sequencing.

### Expression of His-tagged proteins in *E. coli*

*E. coli* BL21(DE3)pLysS competent cells, which were transfected with the pET28b derived plasmids, were grown overnight in Luria broth which contained 50 μg/ml kanamycin. The overnight culture was used to inoculate (1:100) LB medium supplemented with kanamycin (50 μg/ml) and grown at 37 °C until the OD_600_ reached 0.7. Synthesis of the His-tagged protein was initiated by adding isopropyl β-D-1-thiogalactopyranoside (IPTG) to the culture at a final concentration of 0.5 mM. After an overnight incubation in the presence of IPTG at 22 °C, the bacteria were harvested and lysed in BugBuster protein extraction reagent (Novagen) which was supplemented with 10 mM imidazole and 300 mM NaCl. His-tagged proteins were purified under native conditions on Ni-NTA resin (QIAGEN). The proteins were then eluted with elution buffer (50 mM NaH_2_PO_4_, pH 8.0, 300 mM NaCl, and 250 mM imidazole).

For expression of the His-tagged NAOD from the pETDuet-1 His-tagged NAOD-GAPDH vector, the same procedure was used except that the bacteria were grown in Luria broth which contained 100 μg/ml ampicillin.

### Halotag pull-down assay

Crude trophozoite lysate was incubated with HaloLink resin in binding buffer (100 mM Tris, pH 7.6, 150 mM NaCl, and 0.05% IGEPAL (CA630, Sigma) for 60 minutes at room temperature (RT). The HaloLink resin was then washed with buffer (100 mM Tris (pH 7.6), suspended in 1X SDS loading buffer, and boiled for five minutes. The resin was removed from the sample by centrifugation (five minutes @12000 rpm). The proteins in the supernatant were separated on a 10% SDS-PAGE gel and then stained with silver.

### Ethics statement

Polyclonal NAOD antibodies were produced in male BALB/c mice using the following protocol, which was approved by the Technion’s Animal Care and Use Committee (IL-001-01-2010). The Technion-Israel Institute of Technology conforms to Israeli law on the “Prevention of Cruelty to Animals and Experiments on Animals, 5761–2001”. We abide by the rules established by a National Council on Experiments on Animals. Our fundamental standards for animal care and use in research are those defined by the Israeli Animal Welfare Law and those of the American NRC guide (which has a legal status in Israel).

### Production of NAOD antibody

Male BALB/c mice were injected intraperitoneally with 100 μg recombinant His-tagged NAOD protein that was emulsified in complete Freund’s adjuvant. Two and four weeks later, the mice were injected with 100 μg of His-tagged NAOD protein in incomplete Freund’s adjuvant. One week after the 4-week injection, the mice were anesthetized using isoflurane and their blood was collected by cardiac puncture. Serum that was obtained from mice that were not injected with His-tagged NAOD was used as the control.

### Viability of NAT that were exposed to metronidazole

NAT (1 × 10^6^) were incubated in Diamond’s TYI-S-33 medium which contained different concentrations of metronidazole (0–18 μM) for 24 hours. At the end of the treatment, they were transferred to fresh Diamond’s TYI-S-33 medium and cultivated for an additional 24 hours. NAT were counted in 1 ml of phosphate buffer saline (PBS) and their viability was determined by their ability to exclude eosin dye (0.1% final concentration).

### Viability of NAT exposed to OS

NAT (1 × 10^6^) were incubated in Diamond’s TYI-S-33 medium which contained different concentrations of H_2_O_2_ (0–2 mM) for one hour at 37 °C and their viability was determined by their ability to exclude eosin dye (0.1% final concentration).

### Amebicidal activity of activated macrophages

The amebicidal activity of activated macrophages was determined using a previously described protocol^69^. Briefly, RAW 264.7 murine macrophages in DMEM were activated by 22 h of incubation with lipopolysaccharide (LPS) (50 ng/ml), INF-γ (300 u/μl, Prospec), and L-arginine (1 mM, Sigma). Activated macrophages (2 × 10^6^/ml) and *E. histolytica* trophozoites (2 × 10^4^/ml) were co-incubated in DMEM at 37 °C for 6 h. The viability of trophozoites was measured by determining the ability of trophozoites to *exclude eosin dye* (0.1% final concentration).

### Adhesion assay

The adhesion of trophozoites to HeLa cell monolayers was measured using a previously described protocol^70^. Briefly, trophozoites (2 × 10^5^) were washed twice with DMEM without serum, added to wells that contained fixed HeLa monolayers in 1 ml of DMEM without serum, and incubated for 30 minutes at 37 °C. The number of adherent trophozoites was determined by counting the number of trophozoites that remained attached to the HeLa cells after gentle decanting (twice) of the non-adherent trophozoites with warm (37 °C) DMEM under a light microscope.

### RNA extraction, cDNA library preparation and sequencing

Total RNA was extracted from control trophozoites (two biological replicates) and from NAT (three biological replicates) using the TRI reagent kit in accordance with the manufacturer’s instructions (Sigma-Aldrich).

RNA-Seq libraries were prepared using the TruSeq RNA library preparation kit V2 (Illumina Inc. San Diego, CA, USA) according to the manufacturer’s instructions. The libraries were sequenced at the Technion’s Genome Center, Haifa, Israel using the HiSeq 2500 platform (Illumina Inc. San Diego, CA, USA) on 100 single read high output run mode.

### Quality control and analysis of differential gene expression

The quality of the sequenced data was checked using FASTQC v0.10.1 software. The number and percentage of reads that passed through the filter, the control mapping, the error rate, and the per-base quality scores demonstrate that the run was of high-quality (Phred quality scores ≥ 30). The sequenced reads were submitted to GenBank GEO database (http://www.ncbi.nlm.nih.gov/geo/query/acc.cgi?token=gpgfwooqthwxfkl&acc=GSE77044). After quality assessment, the high-quality reads were aligned to the Entamoeba reference genome (http://amoebadb.org/amoeba/) by TopHat version 2.0.11, allowing up to three mismatches per read.

Only uniquely mapped reads were counted for further analysis, using HTSeq-count package version 0.6.1 with ‘union’ mode (http://www-huber.embl.de/users/anders/HTSeq/doc/count.html). Normalization and differential gene expression analysis were performed using the DESeq2 package version 1.6.2. DESeq2 performs statistical tests for determining when a gene is differentially expressed between a pair of conditions. We used a false discovery rate of ≤0.001 and the absolute value of log2 ratio ≥ 1 as the threshold to evaluate the significance of gene expression difference.

### PANTHER classification system

The online PANTHER Version 11.0 (http://pantherdb.org/) was used in this study. Up and down regulated genes in NAT were classified by using the “protein class” ontology setting, the pie chart option and the percent of gene hit against total # Protein Class hits setting. The statistical overrepresentation test was performed with the default setting, the annotation data set corresponding to PANTHER protein class and the Bonferroni correction for multiple testing options selected.

### Quantitative real-time (qRT) PCR-based analysis of gene expression

Total RNA was extracted from trophozoites using a TRI reagent solution (Sigma) and the amount of RNA was quantified by spectrophotometry. Reverse transcription was performed using the High-Capacity cDNA Reverse Transcriptase Kit (Applied Biosystem), according to the manufacturer’s instructions. qRT-PCR was performed using the ABI PRISM 7000 Sequence Detection System and SYBR green PCR Master Mix (Applied Biosystems, Foster City, CA, USA). The PCR reaction mix (10 μl) comprised 5 μl SYBR green master mix, 2.5 μl of diluted (1:5) cDNA and a final concentration of 0.3 μM of each primer. PCR conditions were an initial denaturation step at 95 °C for ten minutes, 40 cycles of denaturation at 95 °C for 15 seconds, and hybridization at 60 °C for 30 seconds.

### Determination of NAOD activity

NAOD activity was determined using a previously described protocol^71^.

### Determination of GAPDH activity

GAPDH activity was determined using a previously described protocol^62^.

### Western blotting

Total protein extracts of *E. histolytica* trophozoites were prepared according to a previously described protocol^72^. Preparation of insoluble protein in *E. histolytica* trophozoites was prepared according to a previously described protocol^73^. Proteins (40 μg) in the extract were resolved on a 10% SDS-PAGE in SDS-PAGE running buffer (25 mM Tris, 192 mM glycine, 0.1% SDS). The resultant protein bands were visualized after staining with Coomassie blue. Alternatively, proteins were electrotransferred in protein transfer buffer (25 mM Tris, 192 mM glycine, 20% methanol, pH 8.3) to nitrocellulose membranes (Whatman, Protran BA83). The blots were first blocked using 3% skim milk, and then probed with 1:1000 mouse polyclonal NAOD antibody, 1:1000 rabbit polyclonal GAPDH antibody (Santa Cruz Biotechnology sc-25778), or 1:500 rabbit polyclonal Gal/GalNAc lectin antibody (a kind gift from N. Guillen, Pasteur Institute, Paris, France) for 16 hours at 4 °C. After incubation with one of the previously described primary antibodies, the blots were incubated with 1:5000 secondary antibody for one hour at RT (Jackson ImmunoResearch), and then developed using enhanced chemiluminescence (SuperSignal West Pico Chemiluminescent Substrate, ThermoFisher Scientific).

### Immunofluorescence microscopy

*E. histolytica* trophozoites were transferred to microscope slides with 8-mm diameter wells, and then incubated for 15 minutes at 37 °C in order to allow them to adhere to the glass surface. The attached trophozoites were washed two times in warm (37°C) PBS, fixed with methanol for 10 minutes at −20 °C, washed again three times in PBS, and then permeabilized with FACS buffer (2% bovine serum albumin in PBS that was supplemented with 0.1% tween) for ten minutes at RT. The slides were then blocked with 5% donkey serum in FACS buffer for 15 minutes at RT. The samples were then probed with either 1:1000 polyclonal mouse EhNAOD antibody only or with 1:1000 rabbit polyclonal GAPDH antibody (sc-25778) only, or with both antibodies. At the end of the incubation, the slides were washed three times in FACS buffer, and then incubated with a 1:250 Indocarbocyanine (Cy3)-conjugated IgG antibody and/or 1:250 Cyanine (Cy2)-conjugated IgG antibody (Jackson ImmunoResearch) for three hours at 4 °C. At the end of the incubation, the nuclei of the trophozoites were stained with 1:1000 4′,6-diamidino-2-phenylindole (DAPI) (Sigma-Aldrich). The samples were then washed with FACS buffer and mounted onto microscope slides with mounting medium (Dako). The specimens were then examined under a confocal immunofluorescence microscope (ZEISS- LSM510 Meta Laser Scanning System confocal imaging system) with a 63X oil immersion objective.

For HaloTag imaging, trophozoites were incubated in Diamond’s TYI-S-33 medium without serum that was supplemented with TMR ligand (Promega) to a final concentration of 1 μM for five minutes at 37 °C. Trophozoites were washed three times with PBS that contained 2% glucose and incubated for one hour at 37 °C in Diamond’s TYI-S-33 medium without serum. Trophozoites were then fixed with methanol and the nuclei of the trophozoites were stained with 1:1000 4′,6-diamidino-2-phenylindole (DAPI) (Sigma-Aldrich). The specimens were then examined under a confocal immunofluorescence microscope with a 63X oil immersion objective.

For quantification of colocalization, specific regions of singly labeled cells were selected first to set the thresholds. Then selected regions of interest were used for pixel quantification. Colocalization of NAOD and GAPDH was quantified using Zeiss Zen 2012 software, which calculates overlap and colocalization coefficient as derived from Pearson’s correlation coefficient. The values for the overlap coefficient range from 0 to 1. An Overlap Coefficient with a value of 1 represents perfectly colocalized pixels.

### HPLC analysis of intracellular amino acids

The measurement of intracellular amino acids concentrations was performed using a previously described protocol^20^.

### ^1^H-NMR spectroscopic analysis of putrescine and acetate in trophozoite lysates

The measurement of intracellular putrescine and acetate concentrations was done using a previously described protocol^20^.

### Structural modeling of NAOD, mNAOD, and GAPDH

The structural model of NAOD was built using the crystal structure of acetylornithine deacetylase of *Rhodopseudomonas palustris* (RPA2325) which is available at the Joint Center for Structural Genomics (http://sbkb.org/center/joint-center-for-structural-genomics). The model was built using the Phyre server (http://www.sbg.bio.ic.ac.uk/phyre2/html/page.cgi?id=index). The coordinates for zinc were obtained by superimposing the model to the template. The structural model of mNAOD was built using the same protocol that was described for building the structural model of NAOD. The structural model of GAPDH was built using the crystal structure of GAPDH of *Leishmania mexicana*^33^. GAPDH from *E. histolytica* and *L. mexicana* share 59% identity. The model was built with a 100% confidence score. The structural superimposition and further structural analysis including image generation, calculation of the solvent accessible surface area, and the Adaptive Poisson-Boltzmann Solver calculations were done using Pymol software (https://www.pymol.org/).

### Protein-Protein Docking Simulations

The final NAOH and GAPDH models obtained from the Phyre server were subjected to energy minimization using AMBER14 software package (http://ambermd.org/). Molecular docking simulations of these low energy models were then done using Cluspro server (http://cluspro.bu.edu/login.php) and the Z-dock server (http://zdock.umassmed.edu/). The docking module of the Cluspro server uses 70,000 rotations to the ligand molecule and scores for each translation of the ligand. The top 1000 structures are then greedy clustered with a 9-angstrom C-alpha root mean square deviation (rmsd) radius.

### Statistical analysis

All the data are presented as the mean ± standard deviation (SD). Significant differences between two groups were determined using unpaired Student’s t-test.

## Additional Information

**How to cite this article**: Shahi, P. *et al*. N-acetyl ornithine deacetylase is a moonlighting protein and is involved in the adaptation of *Entamoeba histolytica* to nitrosative stress. *Sci. Rep.* **6**, 36323; doi: 10.1038/srep36323 (2016).

**Publisher’s note:** Springer Nature remains neutral with regard to jurisdictional claims in published maps and institutional affiliations.

## Supplementary Material

Supplementary Information

Supplementary Information

Supplementary Information

Supplementary Information

Supplementary Information

## Figures and Tables

**Table 1:** Oligonucleotides used in this study.

Primer Name	Sequence	Direction	Restriction site-underline
NAOD	ATGTCTACAACTCTGGGTTATG	Sense	
NAOD KpnI	GGTACCATGTCTACAACTCTGGG	Sense	KpnI
NAOD BamHI	GGATCCTCTTCCCTTTAATGCCG	Antisense	BamHI
NAOD NcoI	CCATGGCTACAACTCTGGGTTAT	Sense	NcoI
NAOD XhoI	CTCGAGTCTTCCCTTTAATGCCG	Antisense	XhoI
NAOD BamHI	GGATCCATGTCTACAACTCTGGGTTATG	Sense	BamHI
NAOD RT	ATGTCTACAACTCTGGGT	Sense	
NAOD RT	TCAACAATTAAGCCAAGG	Antisense	
ArgE NcoI	CCATGGAAAAACAAATTACCG	Sense	NcoI
ArgE XhoI	CTCGAGATGCCAGCAAAAATGGTG	Antisense	XhoI
EcorV GAPDH	GATATCATGTCAATTAAGGTCGGTA	Sense	EcoRV
Kpn1 GAPDH	GGTACCGTGAACTTTAGAAATG	Antisense	KpnI
nat 5′ KpnI	GGTACCATGTCTACTTTGGATGAT	Sense	KpnI
nat 3′ BamHI	GGATCCTTATGGACATGGCATAG	Antisense	BamHI
Actin 5′XhoI	CTCGAGTAGAATTCAAATGATGCTATATTTTGTTC	Sense	XhoI
Actin 3′XhoI	CTCGAGCTCCATATGTACTTTGTATTTCTGTTTCT	Antisense	XhoI
rDNA5′	TCAAAAAGCAACGTCGCTA	sense	
rDNA3′	AGCCCGTAAGGTGATTTCT	antisense	
