# Corrigendum: N-acetyl ornithine deacetylase is a moonlighting protein and is involved in the adaptation of *Entamoeba histolytica* to nitrosative stress

**DOI:** 10.1038/srep45802

**Published:** 2017-04-05

**Authors:** Preeti Shahi, Meirav Trebicz-Geffen, Shruti Nagaraja, Rivka Hertz, Sharon Baumel-Alterzon, Karen Methling, Michael Lalk, Mohit Mazumder, Gourinath Samudrala, Serge Ankri

Scientific Reports 6: Article number: 3632310.1038/srep36323; published online: 11 03 2016; updated: 04 05 2017

The original version of this Article contained errors in the spelling of the author Sharon Baumel-Alterzon, which was incorrectly given as Sharon Alterzon-Baumel. These errors have now been corrected in the PDF and HTML versions of the Article.

