# Supplementary material for: N-acetyl ornithine deacetylase is a moonlighting protein and is involved in the adaptation of Entamoeba histolytica to nitrosative stress
Source: Sci Rep. 2016 Nov 3;6:36323. doi: 10.1038/srep36323 (PMC5093748; doi:10.1038/srep36323)
Supplement: Supplementary Information [file srep36323-s1.doc]

N-acetyl ornithine deacetylase is a moonlighting protein and is involved in the adaptation of *Entamoeba histolytica* to nitrosative stress

Preeti Shahi, Meirav Trebicz-Geffen, Shruti Nagaraja, Rivka Hertz, Sharon Alterzon-Baumel, Karen Methling, Michael Lalk, Mohit Mazumder, Gourinath Samudrala and Serge Ankri

**Supporting information legends**

### Supporting information S1. Expression ratios of control trophozoites vs NAT analyzed by RNA-Seq. The gene expression table contains the following columns: Gene ID, Gene name, Fold change – the fold change between the pair of samples. p-value – p-value for differential expression

**Supporting information S2.** Proteomics analysis of a 35kDa protein that binds to HaloTag-NAOD.

**Supporting information S3.** Proteomics analysis of a NAOD and GAPDH after their co-expression in *E.coli* and their co-purification.

**Supplementary information S4.** Full images of cropped blots or SDS-PAGE gel stained with Coomassie Blue.

(A) Full blot for Figure 3D. (B) Full image for SDS-PAGE gel stained with Coomassie Blue for Figure 5A. (C) Full blot for Figure 7A.
