# Supplementary material for: N-acetyl ornithine deacetylase is a moonlighting protein and is involved in the adaptation of Entamoeba histolytica to nitrosative stress
Source: Sci Rep. 2016 Nov 3;6:36323. doi: 10.1038/srep36323 (PMC5093748; doi:10.1038/srep36323)
Supplement: Supplementary Information [file srep36323-s5.pdf]

N-acetyl ornithine deacetylase is a moonlighting protein and is involved in the adaptation of *Entamoeba histolytica* to nitrosative stress

Preeti Shahi, Meirav Trebicz-Geffen, Shruti Nagaraja, Rivka Hertz, Sharon Alterzon-Baumel, Karen Methling, Michael Lalk, Mohit Mazumder, Gourinath Samudrala and Serge Ankri

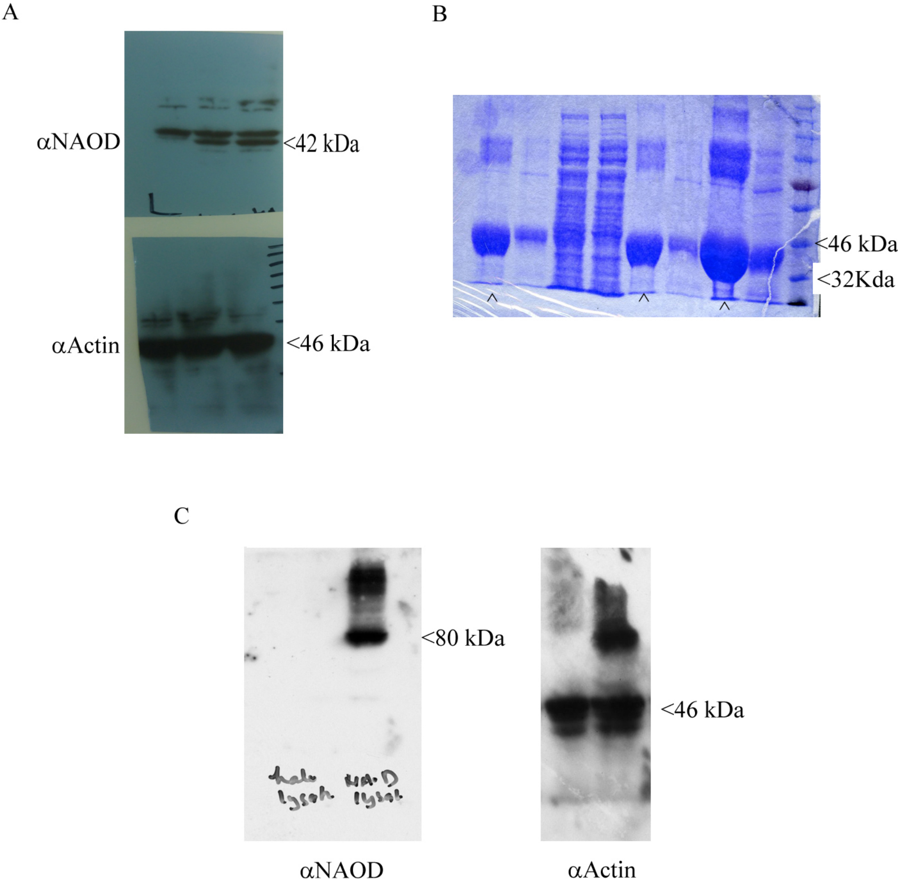
A
αNAOD
<42 kDa
αActin
<46 kDa
B
<46 kDa
<32Kda
C
<80 kDa
<46 kDa
αNAOD
αActin
